# Poly[1,4-bis­(ammonio­meth­yl)cyclo­hexane [di-μ-bromido-dibromido­plumbate(II)]]

**DOI:** 10.1107/S1600536810016806

**Published:** 2010-05-15

**Authors:** Matthew Kyle Rayner, David Gordon Billing

**Affiliations:** a Molecular Sciences Institute, School of Chemistry, University of the Witwatersrand, Private Bag 3, PO Wits 2050, South Africa

## Abstract

The title compound, {(C_8_H_20_N_2_)[PbBr_4_]}_*n*_, crystallizes as an inorganic–organic hybrid with alternating layers of diammonium cations and two-dimensional corner-sharing PbBr_6_ octa­hedra extending parallel to the *bc* plane, which are eclipsed relative to one another. Both PbBr_6_ octa­hedra and the organic cation exhibit 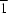 symmetry. The cations inter­act *via* N—H⋯Br hydrogen bonding in the right-angled halogen sub-type of the terminal halide hydrogen-bonding motif.

## Related literature

For hydrogen-bonding nomenclature for inorganic–organic hybrids, see: Mitzi (1999 ▶). Hybrid structures containing diammonium cations have been synthesized by Dobrzycki & Woźniak (2008 ▶) and Zhu *et al.* (2003 ▶). The semiconducting properties of similar hybrids were demonstrated by Mitzi (2004 ▶). For the related chloridoplumbate(II), see: Rayner & Billing (2010*a* ▶) and for the isotypic iodidoplumbate(II), see: Rayner & Billing (2010*b* ▶).
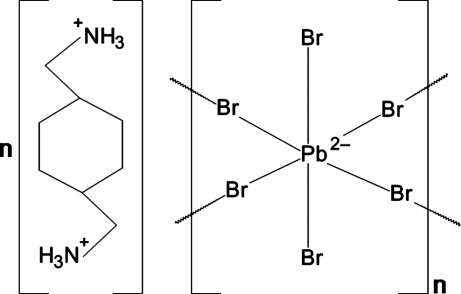


## Experimental

### 

#### Crystal data

(C_8_H_20_N_2_)[PbBr_4_]
*M* *_r_* = 671.09
Monoclinic, 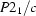

*a* = 12.1042 (6) Å
*b* = 8.1955 (4) Å
*c* = 8.2160 (4) Å
β = 95.693 (1)°
*V* = 811.01 (7) Å^3^
*Z* = 2
Mo *K*α radiation
μ = 20.23 mm^−1^
*T* = 173 K
0.20 × 0.14 × 0.02 mm

#### Data collection

Bruker APEXII CCD area-detector diffractometer
Absorption correction: integration (*XPREP*; Bruker, 2005 ▶) *T*_min_ = 0.091, *T*_max_ = 0.656
10495 measured reflections
1966 independent reflections
1742 reflections with *I* > 2σ(*I*)
*R*_int_ = 0.063

#### Refinement

*R*[*F* ^2^ > 2σ(*F* ^2^)] = 0.021
*wR*(*F* ^2^) = 0.047
*S* = 0.81
1966 reflections
70 parameters
H-atom parameters constrained
Δρ_max_ = 0.78 e Å^−3^
Δρ_min_ = −2.31 e Å^−3^

### 

Data collection: *APEX2* (Bruker, 2005 ▶); cell refinement: *SAINT* (Bruker, 2005 ▶); data reduction: *SAINT*; program(s) used to solve structure: *SHELXS97* (Sheldrick, 2008 ▶); program(s) used to refine structure: *SHELXL97* (Sheldrick, 2008 ▶); molecular graphics: *ORTEP-3 for Windows* (Farrugia, 1997 ▶) and *DIAMOND* (Brandenburg, 1999 ▶); software used to prepare material for publication: *WinGX* (Farrugia, 1999 ▶) and *PLATON* (Spek, 2009 ▶).

## Supplementary Material

Crystal structure: contains datablocks I, global. DOI: 10.1107/S1600536810016806/wm2338sup1.cif

Structure factors: contains datablocks I. DOI: 10.1107/S1600536810016806/wm2338Isup2.hkl

Additional supplementary materials:  crystallographic information; 3D view; checkCIF report

## Figures and Tables

**Table 1:** Selected bond lengths (Å)

Pb1—Br2^i^	2.9821 (3)
Pb1—Br2^ii^	2.9886 (3)
Pb1—Br1^i^	3.0054 (4)

Symmetry codes: (i) 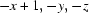; (ii) 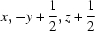.

**Table 2:** Hydrogen-bond geometry (Å, °)

*D*—H⋯*A*	*D*—H	H⋯*A*	*D*⋯*A*	*D*—H⋯*A*
N1—H1*C*⋯Br2^i^	0.91	2.54	3.387 (3)	154
N1—H1*D*⋯Br1^ii^	0.91	2.57	3.378 (3)	148
N1—H1*D*⋯Br2^iii^	0.91	2.94	3.446 (3)	117
N1—H1*E*⋯Br1	0.91	2.60	3.357 (3)	141

Symmetry codes: (i) 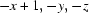; (ii) 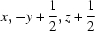; (iii) 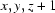.

**Fig. 1.**
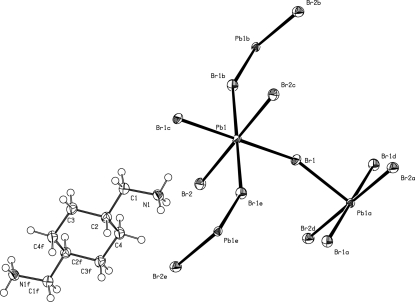
The asymmetric unit of the title compound with atom labels. Displacement ellipsoids were drawn at the 50% probability level. Symmetry codes: (a) 1-x, 1/2+y, 1/2-z (b) 1-x, 1-y, 1-z (c) x, 1/2-y, 1/2+z (d) -x, 1-y, 2-z.

**Fig. 2.**
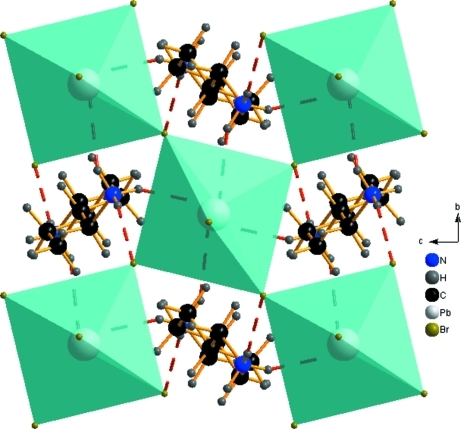
Packing diagram viewed along the a axis. Hydrogen bonds are drawn as dashed red lines.

**Fig. 3.**
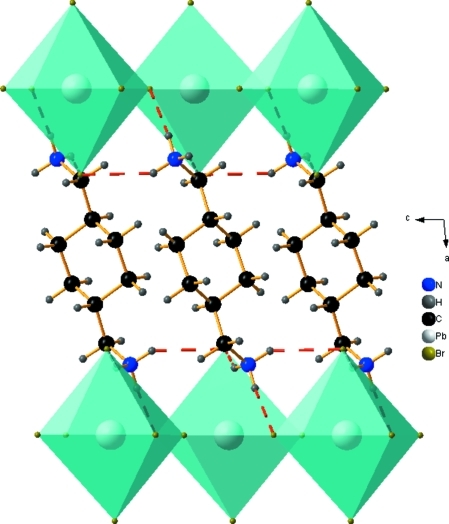
Packing diagram viewed along the b axis. Hydrogen bonds are drawn as dashed red lines.

## supplementary crystallographic information

### Comment

Inorganic-organic hybrid materials are of interest due to their electronic and fluorescent properties (Mitzi, 2004). The title structure (Fig. 1) is one of three 2-dimensional hybrid structures that we have synthesized encorporating this diammonium cation. The structures differ in terms of their halogen ligands, which include bromide (presented here), chloride (Rayner & Billing, 2010*a*) and iodide (Rayner & Billing, 2010*b*). The bromide and iodide hybrids crystallize in the monoclinic crystal system in space group *P*2_1_/*c* while the chloride compound crystallizes in the orthorhombic *Pnma* system.

In the title structure the lead atoms in the PbBr_6_ octahedra occupy inversion centers, giving the octahedra 1 symmetry. The PbBr_6_ octahedra share corners to form layers extending parallel to the *bc* plane. Octahedra from alternate layers are eclipsed relative to one another (Fig. 2). In all three structures only the *trans* form of the cation has been observed giving the cation 1 symmetry (Fig. 3). Very few inorganic-organic hybrid structures encorporating diammonium cations have been reported (Dobrzycki & Woźniak, 2008; Zhu *et al.*, 2003). The ammonium cations interact with the inorganic layer via N—H···*X* (*X* = Br, I and Cl) hydrogen bonding in the right-angled halogen subtype of the terminal halide hydrogen bonding motif (Mitzi, 1999).

### Experimental

A mixture of 0.050 g (0.14 mmol) PbBr_2_ and 0.021 g (0.15 mmol) 1,4-bis-(aminomethyl)-cyclohexane (mixture of isomers) was dissolved in 5 ml HBr at 383 K and slowly cooled at a rate of 0.069 K/min to yield colourless, plate-shaped single crystals suitable for X-ray analysis.

### Refinement

The H atoms on the diammonium cation were refined using a riding-model, with C—H = 0.99 Å, N—H = 0.91 Å and with U_iso_(H)=1.2*U*_eq_(C) or 1.5*U*_eq_(N). The highest residual electron density peak (0.78 e Å-3) was 0.923Å from Pb1.

### Crystal data

**Table:**

(C_8_H_20_N_2_)[PbBr_4_]	*F*(000) = 608
*M**_r_* = 671.09	*D*_x_ = 2.748 Mg m^−^^3^
Monoclinic, *P*2_1_/*c*	Mo *K*α radiation, λ = 0.71073 Å
Hall symbol: -P 2ybc	Cell parameters from 5293 reflections
*a* = 12.1042 (6) Å	θ = 3.0–28.2°
*b* = 8.1955 (4) Å	µ = 20.23 mm^−^^1^
*c* = 8.2160 (4) Å	*T* = 173 K
β = 95.693 (1)°	Plate, colourless
*V* = 811.01 (7) Å^3^	0.20 × 0.14 × 0.02 mm
*Z* = 2	

### Data collection

**Table:**

Bruker APEXII CCD area-detector diffractometer	1966 independent reflections
Radiation source: fine-focus sealed tube	1742 reflections with *I* > 2σ(*I*)
graphite	*R*_int_ = 0.063
φ and ω scans	θ_max_ = 28.0°, θ_min_ = 1.7°
Absorption correction: integration (*XPREP*; Bruker, 2005)	*h* = −15→15
*T*_min_ = 0.091, *T*_max_ = 0.656	*k* = −10→10
10495 measured reflections	*l* = −10→10

### Refinement

**Table:**

Refinement on *F*^2^	Primary atom site location: structure-invariant direct methods
Least-squares matrix: full	Secondary atom site location: difference Fourier map
*R*[*F*^2^ > 2σ(*F*^2^)] = 0.021	Hydrogen site location: inferred from neighbouring sites
*wR*(*F*^2^) = 0.047	H-atom parameters constrained
*S* = 0.81	*w* = 1/[σ^2^(*F*_o_^2^) + (0.0349*P*)^2^ + 0.0549*P*] where *P* = (*F*_o_^2^ + 2*F*_c_^2^)/3
1966 reflections	(Δ/σ)_max_ = 0.011
70 parameters	Δρ_max_ = 0.78 e Å^−^^3^
0 restraints	Δρ_min_ = −2.31 e Å^−^^3^

### Special details

**Table:**

Experimental. Numerical intergration absorption corrections based on indexed crystal faces were applied using the XPREP routine (Bruker, 2005)
Geometry. All esds (except the esd in the dihedral angle between two l.s. planes) are estimated using the full covariance matrix. The cell esds are taken into account individually in the estimation of esds in distances, angles and torsion angles; correlations between esds in cell parameters are only used when they are defined by crystal symmetry. An approximate (isotropic) treatment of cell esds is used for estimating esds involving l.s. planes.
Refinement. Refinement of *F*^2^ against ALL reflections. The weighted *R*-factor wR and goodness of fit *S* are based on *F*^2^, conventional *R*-factors *R* are based on *F*, with *F* set to zero for negative *F*^2^. The threshold expression of *F*^2^ > σ(*F*^2^) is used only for calculating *R*-factors(gt) etc. and is not relevant to the choice of reflections for refinement. *R*-factors based on *F*^2^ are statistically about twice as large as those based on *F*, and *R*- factors based on ALL data will be even larger.

### Fractional atomic coordinates and isotropic or equivalent isotropic displacement parameters (Å^2^)

**Table:**

	*x*	*y*	*z*	*U*_iso_*/*U*_eq_	
C1	0.2360 (3)	−0.0472 (4)	0.4803 (5)	0.0262 (8)	
H1A	0.2798	−0.0711	0.5858	0.031*	
H1B	0.2282	−0.1498	0.4166	0.031*	
C2	0.1224 (3)	0.0131 (4)	0.5124 (4)	0.0216 (8)	
H2	0.1325	0.1095	0.5871	0.026*	
C3	0.0636 (3)	−0.1206 (4)	0.6011 (4)	0.0257 (8)	
H3A	0.0558	−0.2192	0.5312	0.031*	
H3B	0.1093	−0.1499	0.7035	0.031*	
C4	0.0501 (3)	0.0661 (5)	0.3589 (4)	0.0247 (7)	
H4A	0.0869	0.1569	0.3058	0.030*	
H4B	0.0419	−0.0261	0.2807	0.030*	
N1	0.2966 (2)	0.0758 (4)	0.3879 (4)	0.0230 (6)	
H1C	0.3644	0.0356	0.3700	0.035*	
H1D	0.3054	0.1695	0.4473	0.035*	
H1E	0.2569	0.0972	0.2904	0.035*	
Br1	0.25157 (3)	0.02969 (4)	−0.01928 (4)	0.02426 (9)	
Br2	0.50023 (3)	0.18890 (4)	−0.31018 (4)	0.02370 (9)	
Pb1	0.5000	0.0000	0.0000	0.01590 (6)	

### Atomic displacement parameters (Å^2^)

**Table:**

	*U*^11^	*U*^22^	*U*^33^	*U*^12^	*U*^13^	*U*^23^
C1	0.024 (2)	0.0232 (17)	0.0322 (19)	0.0000 (15)	0.0076 (16)	0.0042 (14)
C2	0.024 (2)	0.0206 (17)	0.0210 (17)	−0.0015 (13)	0.0039 (15)	−0.0010 (12)
C3	0.0206 (19)	0.0285 (18)	0.0277 (18)	0.0035 (15)	0.0011 (15)	0.0101 (14)
C4	0.0213 (19)	0.0285 (18)	0.0246 (17)	−0.0011 (15)	0.0040 (14)	0.0042 (14)
N1	0.0203 (16)	0.0229 (15)	0.0267 (15)	−0.0014 (12)	0.0065 (12)	−0.0019 (12)
Br1	0.0241 (2)	0.02380 (17)	0.02478 (17)	−0.00282 (13)	0.00175 (14)	−0.00035 (12)
Br2	0.0311 (2)	0.02090 (17)	0.01975 (16)	0.00481 (13)	0.00579 (13)	0.00695 (12)
Pb1	0.02093 (10)	0.01395 (9)	0.01320 (9)	0.00121 (6)	0.00358 (6)	0.00007 (5)

### Geometric parameters (Å, °)

**Table:**

C1—N1	1.497 (4)	C4—H4B	0.9900
C1—C2	1.509 (5)	N1—H1C	0.9100
C1—H1A	0.9900	N1—H1D	0.9100
C1—H1B	0.9900	N1—H1E	0.9100
C2—C3	1.531 (4)	Br1—Pb1	3.0054 (4)
C2—C4	1.526 (5)	Br2—Pb1	2.9821 (3)
C2—H2	1.0000	Br2—Pb1^ii^	2.9886 (3)
C3—C4^i^	1.514 (5)	Pb1—Br2^iii^	2.9821 (3)
C3—H3A	0.9900	Pb1—Br2^iv^	2.9886 (3)
C3—H3B	0.9900	Pb1—Br2^v^	2.9886 (3)
C4—C3^i^	1.514 (5)	Pb1—Br1^iii^	3.0054 (4)
C4—H4A	0.9900		
			
N1—C1—C2	111.7 (3)	H4A—C4—H4B	108.0
N1—C1—H1A	109.3	C1—N1—H1C	109.5
C2—C1—H1A	109.3	C1—N1—H1D	109.5
N1—C1—H1B	109.3	H1C—N1—H1D	109.5
C2—C1—H1B	109.3	C1—N1—H1E	109.5
H1A—C1—H1B	108.0	H1C—N1—H1E	109.5
C1—C2—C3	108.9 (3)	H1D—N1—H1E	109.5
C1—C2—C4	114.0 (3)	Pb1—Br2—Pb1^ii^	152.724 (12)
C3—C2—C4	110.0 (3)	Br2^iii^—Pb1—Br2	180.000 (11)
C1—C2—H2	107.9	Br2^iii^—Pb1—Br2^iv^	89.827 (4)
C3—C2—H2	107.9	Br2—Pb1—Br2^iv^	90.173 (4)
C4—C2—H2	107.9	Br2^iii^—Pb1—Br2^v^	90.173 (4)
C4^i^—C3—C2	111.6 (3)	Br2—Pb1—Br2^v^	89.827 (4)
C4^i^—C3—H3A	109.3	Br2^iv^—Pb1—Br2^v^	180.000 (15)
C2—C3—H3A	109.3	Br2^iii^—Pb1—Br1	90.075 (10)
C4^i^—C3—H3B	109.3	Br2—Pb1—Br1	89.925 (10)
C2—C3—H3B	109.3	Br2^iv^—Pb1—Br1	84.697 (10)
H3A—C3—H3B	108.0	Br2^v^—Pb1—Br1	95.303 (10)
C3^i^—C4—C2	111.3 (3)	Br2^iii^—Pb1—Br1^iii^	89.925 (10)
C3^i^—C4—H4A	109.4	Br2—Pb1—Br1^iii^	90.075 (10)
C2—C4—H4A	109.4	Br2^iv^—Pb1—Br1^iii^	95.303 (10)
C3^i^—C4—H4B	109.4	Br2^v^—Pb1—Br1^iii^	84.697 (10)
C2—C4—H4B	109.4	Br1—Pb1—Br1^iii^	180.000 (13)
			
N1—C1—C2—C3	177.9 (3)	C3—C2—C4—C3^i^	55.8 (4)
N1—C1—C2—C4	54.7 (4)	Pb1^ii^—Br2—Pb1—Br2^iv^	−0.28 (4)
C1—C2—C3—C4^i^	178.5 (3)	Pb1^ii^—Br2—Pb1—Br2^v^	179.72 (4)
C4—C2—C3—C4^i^	−56.0 (4)	Pb1^ii^—Br2—Pb1—Br1	84.42 (3)
C1—C2—C4—C3^i^	178.4 (3)	Pb1^ii^—Br2—Pb1—Br1^iii^	−95.58 (3)

Symmetry codes: (i) −*x*, −*y*, −*z*+1; (ii) −*x*+1, *y*+1/2, −*z*−1/2; (iii) −*x*+1, −*y*, −*z*; (iv) *x*, −*y*+1/2, *z*+1/2; (v) −*x*+1, *y*−1/2, −*z*−1/2.

### Hydrogen-bond geometry (Å, °)

**Table:**

*D*—H···*A*	*D*—H	H···*A*	*D*···*A*	*D*—H···*A*
N1—H1C···Br2^iii^	0.91	2.54	3.387 (3)	154
N1—H1D···Br1^iv^	0.91	2.57	3.378 (3)	148
N1—H1D···Br2^vi^	0.91	2.94	3.446 (3)	117
N1—H1E···Br1	0.91	2.60	3.357 (3)	141

Symmetry codes: (iii) −*x*+1, −*y*, −*z*; (iv) *x*, −*y*+1/2, *z*+1/2; (vi) *x*, *y*, *z*+1.
